# Modelling Landscape-Level Numerical Responses of Predators to Prey: The Case of Cats and Rabbits

**DOI:** 10.1371/journal.pone.0073544

**Published:** 2013-09-09

**Authors:** Jennyffer Cruz, Alistair S. Glen, Roger P. Pech

**Affiliations:** Landcare Research, Lincoln, New Zealand; University of Alberta, Canada

## Abstract

Predator-prey systems can extend over large geographical areas but empirical modelling of predator-prey dynamics has been largely limited to localised scales. This is due partly to difficulties in estimating predator and prey abundances over large areas. Collection of data at suitably large scales has been a major problem in previous studies of European rabbits (*Oryctolagus cuniculus*) and their predators. This applies in Western Europe, where conserving rabbits and predators such as Iberian lynx (*Lynx pardinus*) is important, and in other parts of the world where rabbits are an invasive species supporting populations of introduced, and sometimes native, predators. In pastoral regions of New Zealand, rabbits are the primary prey of feral cats (*Felis catus*) that threaten native fauna. We estimate the seasonal numerical response of cats to fluctuations in rabbit numbers in grassland–shrubland habitat across the Otago and Mackenzie regions of the South Island of New Zealand. We use spotlight counts over 1645 km of transects to estimate rabbit and cat abundances with a novel modelling approach that accounts simultaneously for environmental stochasticity, density dependence and varying detection probability. Our model suggests that cat abundance is related consistently to rabbit abundance in spring and summer, possibly through increased rabbit numbers improving the fecundity and juvenile survival of cats. Maintaining rabbits at low abundance should therefore suppress cat numbers, relieving predation pressure on native prey. Our approach provided estimates of the abundance of cats and rabbits over a large geographical area. This was made possible by repeated sampling within each season, which allows estimation of detection probabilities. A similar approach could be applied to predator-prey systems elsewhere, and could be adapted to any method of direct observation in which there is no double-counting of individuals. Reliable estimates of numerical responses are essential for managing both invasive and threatened predators and prey.

## Introduction

Predator populations are influenced primarily from the bottom up by prey availability [1], [2] and in turn limit or regulate prey populations [3], [4], often with cascading effects on species at lower trophic levels [5]. Modelling how predators and prey interact allows us to predict changes in the abundances of predators and prey, community structure and ecosystem function, and the effects of management intervention. Therefore these interactions have been the subject of extensive empirical and theoretical work [6]–[11]. However, studies of mammalian predator-prey relationships can be challenging due to the difficulty of accurately monitoring animal populations [12]–[14], and of sampling over the large scales relevant to medium to large predators, which have large home ranges and low population densities. The need for estimating numerical responses of predators and prey at the landscape level is becoming more pressing as conservation programmes shift towards managing metapopulations of threatened and invasive species [15].

Cats and rabbits were introduced to New Zealand by European settlers in the late 18^th^ and early 19^th^ century, respectively. Rabbits form a major portion of the diet of feral cats in many areas [16]. Invasive mammalian predators in Australia and New Zealand are thought to exert stronger top-down impacts than native predators, resulting in the extinction or continuing decline of native prey populations [17]–[21]. These impacts appear to be exacerbated by hyperpredation [22] when European rabbits support populations of feral cats and European red foxes in Australia [23]–[26], and feral cats and ferrets *Mustela furo* in New Zealand [27]–[29]. In some circumstances, invasive predators can regulate rabbit populations, e.g. when rabbits are already at low density due to other factors [25], [30]–[33]. In New Zealand, rabbits are thought to be limited by predators only in high rainfall areas (>700 mm). In the lower rainfall pastoral areas of the South Island, favourable conditions allow rabbits to breed rapidly so that predation has little impact on their populations, which are able to reach very high densities even though predators are widespread and common [29], [34].

The decline in mammalian predators (cats and ferrets) following decreases in rabbit density was established by Norbury and McGlinchy [29] for the low rainfall areas of the Otago and Mackenzie pastoral regions in the South Island of New Zealand. However, the authors were cautious about estimating the magnitude of this numerical response due to their reliance on indices of abundance, which often do not reflect true abundance. Norbury [35] also estimated a combined numerical response of mammalian predators (cats and ferrets) to rabbits from two sites in central Otago, but the generality of this response still needs to be determined. We aimed to extend the work of Norbury and McGlinchy [29] and Norbury [35], by taking advantage of recently developed modelling approaches to estimate the seasonal numerical response of cats to fluctuations in rabbit numbers across tens of thousands of hectares of dry grassland–shrubland within the Otago and Mackenzie regions of New Zealand. This is essential for predicting landscape level effects of changes in rabbit or cat populations as a result of management operations. Our approach uses a novel combination of two modelling techniques: one which explicitly estimates seasonal abundance while accounting for varying detection probability, and the second, which describes seasonal population dynamics while accounting for environmental stochasticity. This new approach can be extended to other predator-prey systems.

## Materials and Methods

### Ethics Statement

Cats and rabbits were counted using spotlighting, a procedure that does not require an animal ethics permit. Spotlighting occurred on over 60 private properties with permission from the land managers/owners. No protected species were sampled in this study.

### Study Areas and Data Collection

**Figure 1:**
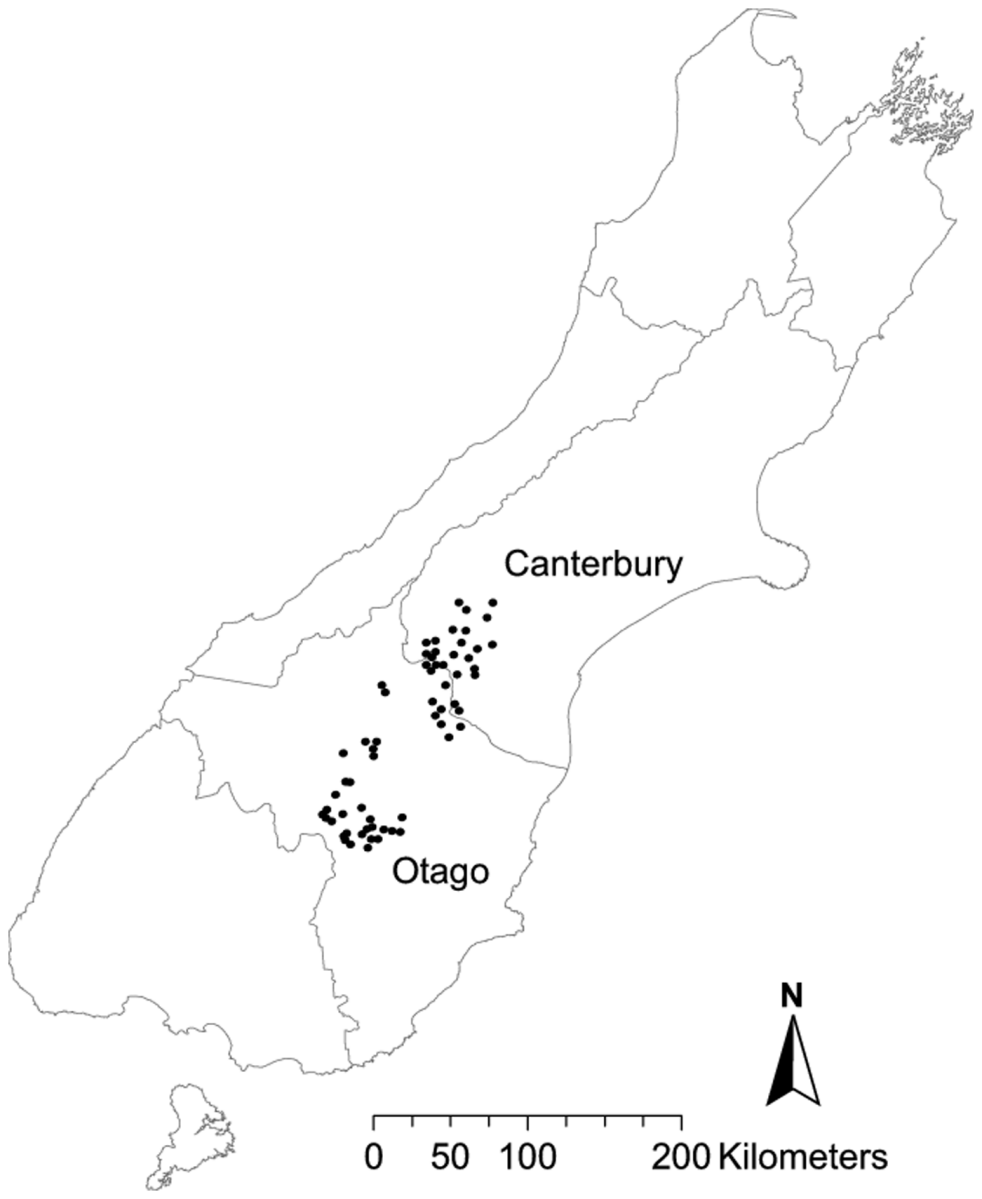
Locations of spotlight transects within the major pastoral regions of the South Island of New Zealand.

Data were compiled from 1990 to 1995 for 66 transects across pastoral regions in Otago (*n* = 30) and the Mackenzie Basin (Southern Canterbury) (*n* = 36), South Island, New Zealand (Fig. 1). In these regions pastures cease growth during winter so rabbit recruitment is highly seasonal, with most young emerging between spring and autumn [34]. This coincides with the breeding period of cats (i.e. spring and summer, sometimes extending into autumn; [36]). In 1991, the New Zealand government implemented the ‘Rabbit and Land Management Programme’ to reduce damage by rabbits to pastoral production. The programme began with two years of large-scale rabbit control through aerial baiting with 1080 (sodium fluoroacetate) poison, which caused an immediate drop in rabbit numbers. The aerial operation was later followed by various forms of ground control, although the intensity and scale were patchy. From 1994, farmers carried out independent, *ad hoc* control. Cats were not targeted for control.

Each transect was sampled by experienced field staff (usually the same observer) from regional councils on two consecutive nights (or sometimes a few days apart), usually twice a year. Sampling occurred between late summer (February) and late spring (November) each year. Each sampling night, an observer counted the number of rabbits and cats seen under a spotlight from a motorcycle driven at a steady speed of about 15 km h^–1^. Further details of protocol for spotlight counts are provided by Parkes *et al.* [37]. Records were kept of the length of transect covered, which varied between locations and sampling periods (mean = 23·1, SD = 5·9 km).

Transect data were collated according to Pollock’s [38] robust design where at each transect, *i*, the two sampling nights represented *j* repeat surveys, during *k* seasons. We divided each year into two 6-month seasons based on the breeding cycle of cats. The breeding season was early spring to late summer (September–February) and the non-breeding season comprised the other 6 months from autumn to late winter (March–August). Populations sampled along each transect were assumed to be closed to demographic effects (mortality, births, migration) between surveys within a season, but open between seasons [38]. Not all transects were sampled in every season, and some transects were sampled more than once within a season. In the latter case, we used the highest estimate obtained for each species in that period. This provided us with an estimate of the minimum number known to be alive.

### Data Analysis

The seasonal population dynamics of rabbits and cats were evaluated simultaneously, using a state-space Bayesian framework. These framework performs well with missing observations [39], as is the case with our data. We took advantage of additional information from repeated surveys within seasons to estimate both detection probability and abundance. An observation model related the observed counts of cats, 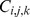, and rabbits, 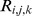, at transect *i*, survey *j* and season *k*, to their abundances, 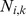, and detection probability, 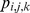, through a binomial process for open populations, within the Bayesian state-space framework [40]. The ability to detect an individual on an entire transect could vary between transects and seasons, and as a result of factors including weather, time of day or search effort [41]. No information was recorded on the weather during the surveys. The time spent surveying each kilometre of transect was standardized by driving at a constant speed, but the length of transect (and thus search effort) varied between locations and seasons. For each species, we modelled 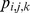 as a logit-linear function with intercepts specific to each season (α*_k_*), coefficients for linear and quadratic effects (β_1_ and β_2_ respectively) of varying transect length (*TL*) on detection, and random observation errors (δ) to account for additional transect- and survey-specific variation in detection. Transect length was standardized to have a mean of zero and unit variance to improve model convergence. The overall observation models for both species are:
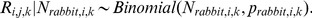
(1)

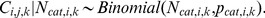
(2)

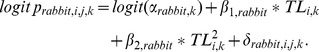
(3)

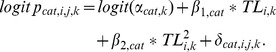
(4)


Uninformative priors used in the observation models are summarized in Appendix S1. The abundance estimates were incorporated into a process model for each species that described their seasonal population dynamics using a Gompertz-type model of density-dependent population dynamics [42], [43] described by:
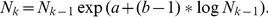
(5)


Here, 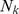 is the abundance of the species at each season, *a* is the intercept, and *b* describes the type of density dependence [43]. A value of *b* = 1 suggests no density dependence, when 0<b<1, decreasing values indicate increasingly strong negative density dependence, and when b<0, increasing absolute values indicate increasingly positive density dependence, which often results in boom-and-bust population trajectories [43]. The Gompertz model for cats and rabbits was modified under a Bayesian framework as follows:
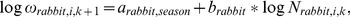
(6)

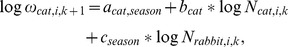
(7)

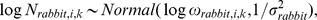


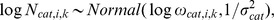



Uninformative priors used in the Gompertz models are summarized in Appendix S1. We converted eqn 5 to the logarithmic scale so that it became a simple linear function for each species (eqns 6 and 7) and added an additional spatial dimension so that population growth was assessed at each transect, *i*, and season (time-step), *k.* The logs of the transect- and season-specific abundances, log *N_i,k_*, are assumed to come from a normal distribution with mean 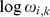 and variance 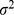, to account for stochasticity (process error) in the population dynamics. The *a_season_* parameter is a seasonal intercept for the breeding and non-breeding seasons. For example, a significant value for the abundance of rabbits measured in the cat breeding season (*a*_b_) indicates a delayed influence of rabbit abundance in the immediately preceding non-breeding season. Density dependence, *b*, is assumed to be the same for breeding and non-breeding seasons (following the approach used by Turchin [44] to adapt the logistic model for annual population dynamics to one for seasonal dynamics). For cats, the process model also incorporates a seasonal parameter, *c_season_*, for the possible numerical response of cats to rabbits. For example, a significant value for the abundance of cats in their non-breeding season (*c*_nb_) indicates a delayed influence of rabbit abundance in the immediately preceding breeding season. The seasonal intercept for rabbits, *a_rabbit,season_*, is equivalent to the intrinsic rate of increase, but not so for cats since *a_cat,season_* is also affected by the additional parameters in the model (i.e. the effect of rabbits) [45]. By using rabbit abundance on the logarithmic scale, the model is robust to assumptions of rate or ratio-dependence of cats to rabbits [45]. In other words, the model does not specify whether rabbits influenced cats through prey dependence or ratio dependence [46]. Top-down effects of cats on rabbit abundance were not modelled, because previous studies suggest predation does not limit rabbits in similar environments in New Zealand [29].

Given the data, model and priors, parameter estimates and 95% Bayesian Credible Intervals (CIs) were obtained through Markov Chain Monte Carlo (MCMC) sampling in WinBUGS (version 1.4.3, http://www.mrc-bsu.cam.ac.uk/bugs/), which we called from R (version 2.13.2, http://cran.r-project.org), via the R2WinBUGS package [47]. The R code is provided in Appendix S2. Model convergence was assessed graphically and with the Gelman–Rubin statistic [48]. The complexity of the models meant that convergence of the chains was slow. Therefore, for each model, we ran three parallel Markov chains for 420 000 iterations, thinned by 10, with the first 400 000 being discarded as burn-in. The thinning and burn-in processes reduced the effects of initial parameter estimates and auto-correlation [49]. Model parameters were judged significant if their 95% CIs did not overlap zero [50]. Abundance estimates for each location were divided by transect length to obtain ‘density’ indices of individuals per km of transect, which were comparable between sites and seasons.

Goodness of fit of the posterior distributions of cat and rabbit abundance estimates was assessed using a version of Bayesian *p*-values that compares abundance estimates simulated under the model (predicted discrepancy) to replicates of the abundance data (realized discrepancy), using a chi-squared statistic [51], [52]. The Bayesian *p*-value is the probability that the predicted abundance estimates are more extreme than the realized estimates. This is expected to occur half the time under perfect fit. Bayesian *p*-values >0·1 and <0·9 were considered good fit [51], [52].

## Results

**Figure 2:**
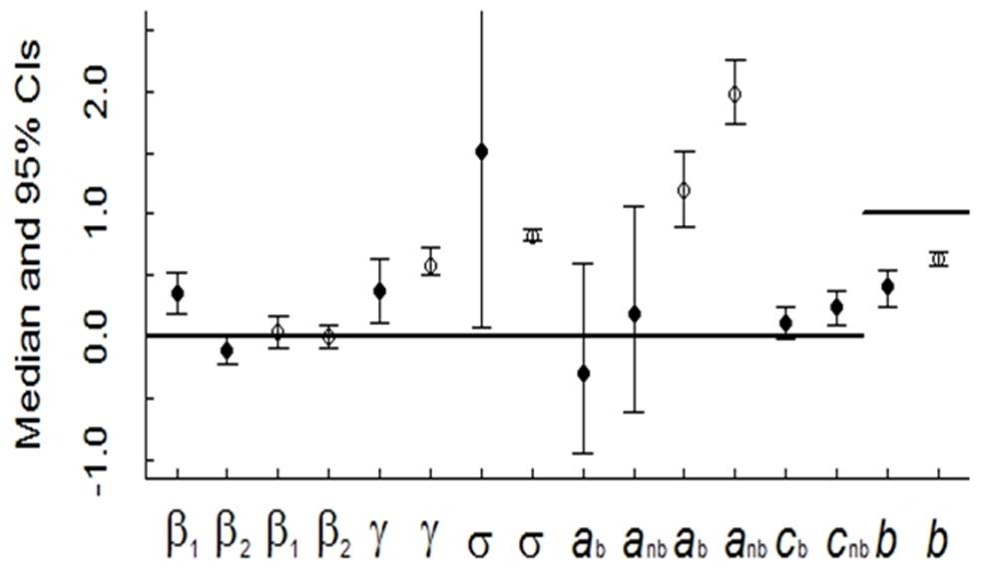
Median and 95% credible intervals (CIs) for parameters in the observation and Gompertz models: cats (filled circles); rabbits (hollow circles). For each species the parameters in the observation model are β_1_, β_2,_ which respectively describe the linear and quadratic effects of transect length on detection, and γ, the observation error. Parameters in the Gompertz model comprise seasonal intercepts, *a*_b_, *a*_nb_ (for the breeding (b) and non-breeding seasons (nb)), a coefficient for density dependence, *b*, and the process error, σ. For cats, the Gompertz model also included seasonal coefficients for the log of rabbit abundance in the previous sampling season, *c*_b_, *c*_nb_. Parameters with 95% CIs overlapping the horizontal lines are non-significant.

**Figure 3:**
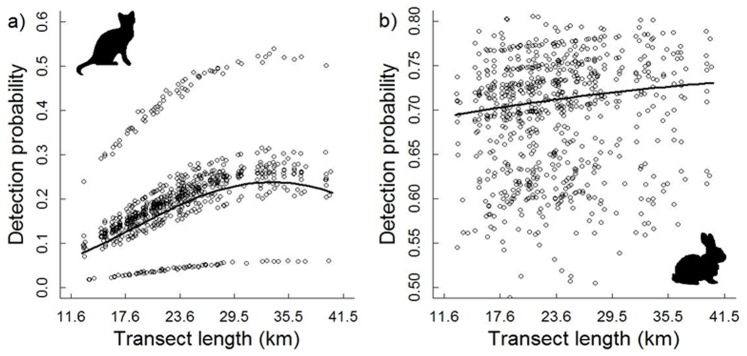
Predicted detection probability for cats and rabbits in relation to transect length. Detection probability for cats (a) increased significantly with increasing transect length up to 25 km. Detection probability for rabbits (b) was not significantly associated with transect length. The black line is the posterior mean across transects and seasons.

The probability of detecting an individual on a transect varied between transects and seasons for cats and rabbits. The significant observation errors (95% CIs for γ not overlapping zero) suggested unexplained survey-specific heterogeneity for cats and rabbits (Fig. 2). Transect length was significantly associated with detecting a cat on a transect: the curvilinear relationship suggested the highest probability of detection was achieved with transect lengths >25 km (Fig. 3a). The probability of detecting a cat on a transect also differed between seasons, and was exceptionally low during the first sampling period (March–August 1990; mean of 0·041 across all transects) and exceptionally high for the fourth period (September–February 1991; mean of 0·45). This is represented in the distinct bands visible in Fig. 3a. The probability of detecting a rabbit on a transect ranged from 0·5 to 0·8, but was not significantly associated with the transect length (Fig. 3b). Overall, spotlighting yielded low detection for cats (mean = 0·20, SD = 0·09) and high detection for rabbits (mean = 0·69, SD = 0·06) (Fig. 3).

**Figure 4:**
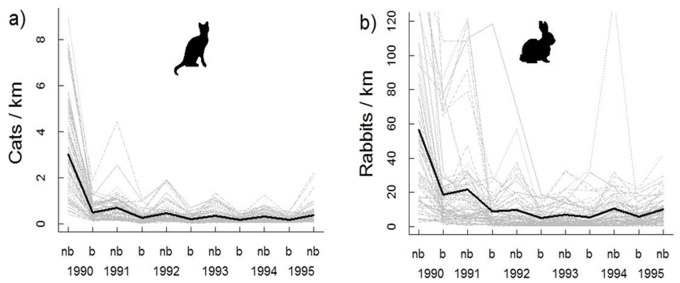
Estimated density (individuals per transect kilometre) of cats and rabbits in the breeding and non-breeding seasons for cats (b and nb respectively). Estimates of cat (a) and rabbit (b) abundance were higher during the non-breeding seasons. The thin grey lines are the mean density estimates for each spotlight transect (n = 66), while the thick black line is the mean across transects.

The Bayesian *p*-values suggested good model fit, with similar discrepancies between the predicted and realized abundance estimates for cats (0·25) and rabbits (0·30). Estimates of cat and rabbit abundance were higher during the non-breeding seasons (Fig. 4). Estimates of rabbit abundance also suggested large declines immediately after 1990 at several sites (coinciding with the large-scale aerial baiting), with populations stabilizing at low numbers for the remainder of the study (Fig. 4b).

**Figure 5:**
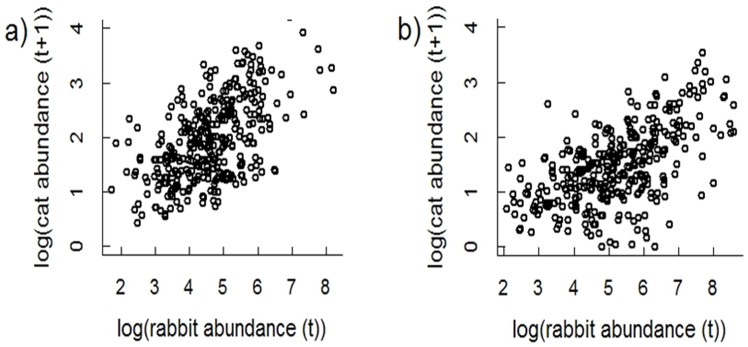
Predicted log(cat abundance) in relation to log(rabbit abundance) in the previous season. Graphs show (a) the non-breeding season (March–August) and (b) the breeding season for cats (September–February). t = time.

In the seasonal Gompertz population growth models, density-dependence (*b*) was significant (*b* ≠ 1) and was strongly negative (0<*b*<1) for both cats and rabbits (Fig. 2). The seasonal intercepts for cats (*a_cat,season_*) were centred at zero and had large overlap between the breeding and non-breeding seasons, indicating substantial uncertainty (Fig. 2). This uncertainty was also reflected in the large CIs associated with the process error for cats (σ_cat_), but not so for rabbits (σ_rabbit_, Fig. 2). The seasonal estimates of the intrinsic rate of increase of rabbits (*a_rabbit,season_*) were significantly different to zero: median values 1·19 (95% CI 0·89–1·51) and 1·98 (95% CI 1·72–2·26) for the cat breeding and non-breeding seasons respectively. This suggests rabbit populations could experience growth during both seasons depending on abundance in the preceding season, with greater increases (based on spotlight counts) possible during the cat non-breeding season (Fig. 2). Cat abundance measured in their non-breeding season was significantly associated with rabbits detected in the preceding season (*c*_nb_, Fig. 2 and Fig. 5). However, cats in the breeding season were not significantly associated with rabbits detected in the preceding season (95% CIs of *c*_b_ overlapped zero, Fig. 2).

## Discussion

Our results show that the abundance of feral cats is driven strongly by that of rabbits across large areas of pastoral New Zealand. Cat populations in their non-breeding season were associated with rabbit abundance measured during the preceding season, but a similar delayed association was not evident for cats during their breeding season. Previous dietary studies in New Zealand show that rabbits are the most significant prey for cats during spring and summer in many areas [16], [53]. Therefore, rabbit abundance almost certainly influenced reproductive rates and juvenile survival of cats, and the time lag before the young cats were detected via spotlighting is the likely explanation for the significant value of the seasonal parameter, *c*_nb_, in the numerical response of cats. Rabbit numbers during autumn and winter had a non-significant effect on cat abundance in the following breeding season. During winter, the rabbit population was likely composed largely of adults, which are more difficult for cats to capture [54]. Over-winter survival of cats may therefore have been influenced not solely by rabbits, but also by the availability of alternative prey including birds, lizards and invertebrates [16], as well as by environmental factors such as extreme minimum winter temperatures and long periods of snow cover. Overall, our results extend previous work from New Zealand [29], [35] and support studies elsewhere, which report that rabbit declines lead to predator declines [55]–[58].

The observation models in the hierarchical analysis suggest that detection probability on a transect for both cats and rabbits varied between transects and seasons. Rabbit detection was much higher than cats regardless of transect length, suggesting that even the shortest transects used (*c*. 12 km) were adequate for sampling rabbits. Conversely, the non-linear relationship between cat detection and transect length suggests that detection improved with longer transects, and levelled off when transects were longer than approximately 25 km. This highlights the need to sample cats using long transects. This result is intuitive as cats, like many other predators, can have large home ranges with limited overlap [59]–[61] so that large areas are likely to be required to obtain meaningful estimates of their abundance. Longer transects may also be required to enumerate rabbit populations in areas where the density is low, such as the Iberian Peninsula [55]. Cat detection on a transect also varied with sampling period. The exceptionally low detection of cats during March–August 1990 was probably because the sampling protocol initially focused on counting rabbits, although after this first sampling session there was an increased focus on counting cats as well as rabbits. Reasons for the exceptionally high detection of cats during September–February 1991 are unknown. The estimated random errors in the detection models for both cats and rabbits suggest that additional factors unaccounted for in our analyses, such as weather conditions, time of day and/or observer effects [40], also influenced detection.

Data used for our analyses pre-dated the release of the rabbit haemorrhagic disease (RHD) virus in New Zealand in 1997 [37]. Our estimates of the intrinsic rate of increase for rabbits, which we estimated for 6-month seasons, are in agreement with the estimates of rate of increase per month by Caley and Morley [14], who had similarly-defined seasons and used data collected from two sites in North Canterbury just before the arrival of RHD. Localized poisoning and other forms of control such as shooting and warren fumigation that were used sporadically by farmers during much of our study period can reduce rabbit populations temporarily [35] but are unlikely to affect rabbits’ intrinsic rate of increase. These control measures are more likely to have masked some consequences of high rabbit abundance for their population dynamics, and hence might have affected our estimate of the parameter for density dependence. In contrast, destruction of warrens typically used for rabbit control in many areas of Australia [62] and/or disease [37], [55], [63] can reduce rabbits to low densities for sustained periods, effectively reducing the capacity of rabbit populations to increase at their maximum rate. Therefore, since 1995 in Australia and 1997 in New Zealand, seasonal rates of increase are likely to have changed due to the impact of RHD, although Parkes et al. [37] suggest its efficacy is waning.

To our knowledge, this is the first study to incorporate mechanistic population models under a binomial mixture framework, as suggested by Kéry and Schaub (p. 387) [52]. Previous studies of predator–prey dynamics have estimated population sizes using mark–recapture and/or radio-telemetry data, which are expensive to obtain [64], or they have used relatively inexpensive measures such as spotlight counts to produce indices of abundance [25], [29], [65]. Such indices represent confounded estimates of abundance and detection probability and rarely have a linear relationship with abundance [66]. As was evident in our data, detection probability can vary widely between transects and seasons, which means indices can produce biased estimates of population trends. Our approach increases the value of spotlight data by delivering explicit estimates of population size for rabbits and cats. This is made possible by repeated sampling within each primary period, which allows estimation of detection probabilities and, in particular, allows for changes in detection probabilities over time [41]. A similar approach could aid in modelling predator–prey dynamics in a wide range of systems. For example, it could be adapted to any method of direct observation such as bird counts [67] or aerial surveys for large herbivores [68], in which there is no double-counting of individual animals. However, the method is not suitable for measures of animal sign such as spoor [69], [70] or for burrow counts [71].

Our results show that populations of an invasive predator (feral cats) across two major pastoral regions in New Zealand’s South Island are influenced by the abundance of invasive prey (rabbits) during spring and summer. This might be expected to intensify predation on secondary prey species (i.e. hyperpredation), including native species of conservation concern [72]. We therefore support previous suggestions that controlling rabbit populations should not only reduce their direct impacts (e.g. herbivory), but also limit the abundance of feral cats [65]. This is likely to have flow-on effects by alleviating predation on native fauna. Cats are recognized as a threat to many native animals in New Zealand [36], including many iconic or endangered birds [73]–[75] and reptiles [21], [35]. However, sudden reductions in rabbit abundance can cause prey switching by cats, intensifying predation on native birds [76] and lizards [35] in the short term. Rabbit control should therefore be accompanied by predator control [35]. An inclusive approach to managing rabbits and introduced predators has also been recommended in other parts of the world [57], [77], [78]. Similarly, concurrent management of predators and alternative prey has been recommended for conservation of rare or endangered prey species such as huemul deer (*Hippocamelus bisulcus*) in Chile and woodland caribou (*Rangifer tarandus caribou*) in Canada [79].

Reliable estimates of the numerical response of predators to prey over large spatial scales are essential to manage metapopulations of invasive and threatened species. Our models for the seasonal numerical responses of cat populations to rabbits extend previous work on cat-rabbit interactions in New Zealand to a landscape scale. Our novel combination of two modelling approaches permits the use of spotlight counts, which are relatively inexpensive to collect and cover areas sufficiently large to detect population trends for wide-ranging animals. This approach can be extended to other predator-prey systems and can incorporate additional extrinsic factors that might influence the abundance of predators and prey.

## Supporting Information

Appendix S1 **Uninformative priors used to model the abundance of rabbits and cats across pastoral regions in Otago and the Mackenzie Basin (Southern Canterbury), South Island, New Zealand.**
(DOCX)

Appendix S2 **R code used to analyse population dynamics of rabbits and cats.**
(DOC)
