# Supplementary material for: Modelling Landscape-Level Numerical Responses of Predators to Prey: The Case of Cats and Rabbits
Source: PLoS One. 2013 Sep 9;8(9):e73544. doi: 10.1371/journal.pone.0073544 (PMC3767736; doi:10.1371/journal.pone.0073544)
Supplement: Appendix S1 — Uninformative priors used to model the abundance of rabbits and cats across pastoral regions in Otago and the Mackenzie Basin (Southern Canterbury), South Island, New Zealand. (DOCX) [file pone.0073544.s001.docx]

**Appendix S1.** Uninformative priors used to model the abundance of rabbits and cats across pastoral regions in Otago and the Mackenzie Basin (Southern Canterbury), South Island, New Zealand.

Uninformative priors in the observation model relating the observed counts of cats and rabbits at transect *i*, survey *j* and season *k*, to their abundances and detection probability, through a binomial process are:

$\alpha_{rabbit,k}\sim Uniform(0,1)$.

$\alpha_{cat,k}\sim Uniform(0,1)$.

$\beta_{rabbit}\sim Normal(0,100)$.

$\beta_{cat}\sim Normal(0,100)$.

$\delta_{rabbit,i,j,k}\sim Normal(0,{1/\gamma}_{rabbit}^{2})$.

$\delta_{cat,i,j,k}\sim Normal(0,{1/\gamma}_{cat}^{2})$.

$\gamma_{rabbit}\sim Uniform(0,3)$.

$\gamma_{cat}\sim Uniform(0,3)$.

Uninformative priors in the Gompertz-type model describing the seasonal population dynamics of rabbits and cats are:

$\sigma_{rabbit}\sim Uniform(0,3)$,

$\sigma_{cat}\sim Uniform(0,3)$,

$a_{rabbit,season}\sim Uniform(-5,5)$,

$a_{cat,season}\sim Uniform(-5,5)$ ,

$b_{rabbit}\sim Uniform(-1,1)$,

$b_{cat}\sim Uniform(-1,1)$,

$c_{season}\sim Normal(0,100)$,

$\omega_{rabbit,i,1}\sim Uniform\left( {maxR}_{i},\left( {maxR}_{i}+1000 \right) \right)$,

$\omega_{cat,i,1}\sim Uniform({maxC}_{i},\left( {maxC}_{i}+100 \right))$.

The ${maxR}_{i}$ and ${maxC}_{i}$ values are the counts of rabbits and cats known to be alive on transect *i*. Because rabbits have persisted throughout the Mackenzie Basin and Otago since the 1870s [[1](#_ENREF_1)], we limited ${maxR}_{i}$ and ${maxC}_{i}$ to ≥ 2, that is, a minimum abundance of two individuals.

**References**

1. Norbury G, Reddiex B (2005) European rabbit *Oryctolagus cuniculus* (Linnaeus, 1758). In: King CM, editor. The Handbook of New Zealand Mammals 2nd Edition. Melbourne: Oxford University Press. pp. 131-150.
