# Supplementary material for: Modelling Landscape-Level Numerical Responses of Predators to Prey: The Case of Cats and Rabbits
Source: PLoS One. 2013 Sep 9;8(9):e73544. doi: 10.1371/journal.pone.0073544 (PMC3767736; doi:10.1371/journal.pone.0073544)
Supplement: Appendix S2 — R code used to analyse population dynamics of rabbits and cats. (DOC) [file pone.0073544.s002.doc]

**Appendix S2.** R code used to analyse population dynamics of rabbits and cats. The code is adapted in part from code in Kéry & Schaub (2012), chapters 5 (p.127) and 12 (p. 405).

Kéry M, Schaub M (2012) Bayesian population analysis using WinBUGS: a hierarchical perspective. Amsterdam, The Netherlands: Elsevier. 535 p.

#################### START OF CODE

memory.limit(4000)

maind<- read.table("finaldata.txt", header=T)

attach(maind)

uRoute<-unique(Route)

nroute <- length(uRoute) # total number of transects (sites)

nseason<- max(Cperiod) #total number of seasons

J<- 2 #sampling days within a season

nonbreed<-seq(1,11,2)

breed<-seq(2,10,2)

#routes had different transect lengths:

transect.length <- matrix(NA,nroute,nseason)

for (k in 1:nseason){

for (i in 1:nroute){

transect.length[i,k]<-Length[Route==uRoute[i]& Cperiod==k][1]

}}

for (i in 1:nroute){

transect.length[i, is.na(transect.length[i,])]<- max(transect.length[i,],na.rm=T)

}

transect.length<-scale(transect.length)

# data set within a robust design framework with sites i, primary sessions k and secondary

# session j

R<- array(NA,dim=c(nroute,J,nseason))

C<- array(NA,dim=c(nroute,J,nseason))

for (i in 1:nroute){

for (k in 1:nseason){

R[i,1,k]<- RabbitD1[Route==uRoute[i] & Cperiod==k][1] #rabbit counts day1

R[i,2,k]<- RabbitD2[Route==uRoute[i] & Cperiod==k][1] #rabbit counts day2

C[i,1,k]<- CatD1[Route==uRoute[i] & Cperiod==k][1] #cat counts day1

C[i,2,k]<- CatD2[Route==uRoute[i] & Cperiod==k][1] #cat counts day2

}}

#max no. of sightings for each site and season:

Rmax<- matrix(1, nrow=nroute, ncol=nseason)

Cmax<- matrix(1, nrow=nroute, ncol=nseason)

for (i in 1:nroute){

for (k in 1:nseason){

Rmax[i,k] <- max(R[i,,k])

Cmax[i,k] <- max(C[i,,k])

}}

Rmax[ is.na(Rmax)]<-1

Cmax[ is.na(Cmax)]<-1

for (i in 1:nroute){

for (k in 1:nseason){

Cmax[i,k]<-ifelse(Cmax[i,k]<2,2,Cmax[i,k])

Rmax[i,k]<-ifelse(Rmax[i,k]<2,2,Rmax[i,k])

}}

#abundance in season 1 for each site:

Rabund1<-matrix(NA, nrow=nroute, ncol=nseason)

Cabund1<-matrix(NA, nrow=nroute, ncol=nseason)

for (i in 1:nroute){

Rabund1[i,1]<-Rmax[i,1]

Cabund1[i,1]<-Cmax[i,1]

}

maxRabund1<-Rabund1[,1]+1000

################################################################################

#Calling WinBUGS from R:

cat("

model { # Start model description

#### Prior distributions

# for process model

for (i in 1:nroute){

w.rabbit[i,1]~dunif(Rabund1[i,1],maxRabund1[i])

w.cat[i,1]~dunif(Cabund1[i,1],100)

}

for (b in 1:2){

R.a[b]~dunif(-1,5) #prior for intrinsic rate of increase for rabbits

C.a[b]~dunif(-1,3) #prior for intrinsic rate of increase for cats

C.c[b]~dnorm(0,0.01) I(-20, 20) #effect of rabbit abundance for cats

}

R.b~dunif(-1,1) #density dependence effect for rabbits

C.b~dunif(-1,1) #density dependence effects for cats

Rsigma.siteint~dunif(0,3)

Rtau.siteint <- 1/(Rsigma.siteint*Rsigma.siteint)

Csigma.siteint~dunif(0,3)

Ctau.siteint <- 1/(Csigma.siteint*Csigma.siteint)

# for observation model

#detection varies between sites and seasons

for(b in 1:2){

R.beta[b]~dnorm(0,0.01) I(-20, 20)

C.beta[b]~dnorm(0,0.01) I(-20, 20)

}

for(k in 1:nseason){

Rp0[k]~dunif(0,1)

Cp0[k]~dunif(0,1)

Rlogitp0[k]<-log(Rp0[k]/(1-Rp0[k])) #logit-scale mean detection prob of rabbits in season k

Clogitp0[k]<-log(Cp0[k]/(1-Cp0[k])) #logit-scale mean detection prob of cats in season k

}

Rtau.lp<-1/(Rsigma.lp*Rsigma.lp)

Ctau.lp<-1/(Csigma.lp*Csigma.lp)

Rsigma.lp~dunif(0,3)

Csigma.lp~dunif(0,3)

#### Ecological and observation models for rabbits

for(i in 1:nroute){ # Loop over sites

for(k in 1:5){ # Loop for nonbreeding and breeding seasons

log(w.rabbit[i,(nonbreed[k]+1)])<- R.a[1] + R.b*logN.rabbit[i,nonbreed[k]]

log(w.rabbit[i,(breed[k]+1)])<-R.a[2] + R.b*logN.rabbit[i,breed[k]]

log(w.cat[i,(nonbreed[k]+1)])<- C.a[1] + C.b*logN.cat[i,nonbreed[k]]

+ C.c[1]*logN.rabbit[i,nonbreed[k]]

log(w.cat[i,(breed[k]+1)])<- C.a[2] + C.b*logN.cat[i,breed[k]]

+ C.c[2]*logN.rabbit[i,breed[k]]

}

for(k in 1:nseason){

logw.rabbit[i,k]<-log(w.rabbit[i,k])

logw.cat[i,k]<-log(w.cat[i,k])

logN.rabbit[i,k]~dnorm(logw.rabbit[i,k],Rtau.siteint)

logN.cat[i,k]~dnorm(logw.cat[i,k],Rtau.siteint)

N.rabbit[i,k]<-round(exp(logN.rabbit[i,k]))

N.cat[i,k]<-round(exp(logN.cat[i,k]))

for(j in 1:J){ # Loop over replicate counts

# Next are the observed data

R[i,j,k] ~ dbin(R.p[i,j,k], N.rabbit[i,k])

C[i,j,k] ~ dbin(C.p[i,j,k], N.cat[i,k])

# Avoid WinBUGS logit function which may cause trouble (K?ry 2010)

R.p[i,j,k] <- exp(R.lp[i,j,k])/(1+exp(R.lp[i,j,k]))

C.p[i,j,k] <- exp(C.lp[i,j,k])/(1+exp(C.lp[i,j,k]))

# Truncation to avoid numerical overflow

R.lp[i,j,k] ~ dnorm(Rmu.lp[i,j,k], Rtau.lp)I(-20, 20) #random error defined implicity

C.lp[i,j,k] ~ dnorm(Cmu.lp[i,j,k], Ctau.lp)I(-20, 20)

Rmu.lp[i,j,k]<- Rlogitp0[k] + R.beta[1]*transect.length[i,k] + R.beta[2]*pow(transect.length[i,k],2)

Cmu.lp[i,j,k]<- Clogitp0[k] + C.beta[1]*transect.length[i,k] + C.beta[2]*pow(transect.length[i,k],2)

#Model fit using Chi-squared discrepancy

#compute fit statistics for observed data

R.eval[i,j,k]<-R.p[i,j,k]*N.rabbit[i,k]

C.eval[i,j,k]<-C.p[i,j,k]*N.cat[i,k]

R.E[i,j,k]<-pow((R[i,j,k]-R.eval[i,j,k]),2)/(R.eval[i,j,k]+0.5)

C.E[i,j,k]<-pow((C[i,j,k]-C.eval[i,j,k]),2)/(C.eval[i,j,k]+0.5)

#generate replicate data & compute fit stats for them

R.new[i,j,k]~dbin(R.p[i,j,k],N.rabbit[i,k])

C.new[i,j,k]~dbin(C.p[i,j,k],N.cat[i,k])

E.Rnew[i,j,k]<-pow((R.new[i,j,k]-R.eval[i,j,k]),2)/(R.eval[i,j,k]+0.5)

E.Cnew[i,j,k]<-pow((C.new[i,j,k]-C.eval[i,j,k]),2)/(C.eval[i,j,k]+0.5)

} # Close j loop

ik.Rp[i,k]<-mean(R.p[i,,k])

ik.Cp[i,k]<-mean(C.p[i,,k])

} # Close k loop

} # Close i loop

#Derived and other quantiles

for (k in 1:nseason){

mean.Nrabbit[k]<-mean(N.rabbit[,k])

mean.Ncat[k]<-mean(N.cat[,k])

mean.Rp[k]<-mean(ik.Rp[,k])

mean.Cp[k]<-mean(ik.Cp[,k])

}

R.fit<-sum(R.E[,,])

C.fit<-sum(C.E[,,])

fit.Rnew<-sum(E.Rnew[,,])

fit.Cnew<-sum(E.Cnew[,,])

}

",file="mod1.txt")

data <- list ("R","nroute","nseason","J",'breed','nonbreed','Rabund1','maxRabund1','transect.length',

'C','Cabund1')

inits <- function (){

list (logN.rabbit=log(Rmax),w.rabbit=Rabund1,R.a=rnorm(2,2,0.1),R.beta=rnorm(2,0,0.1),

Rsigma.lp=runif(1,1,2),R.b=runif(1,-1,1),Rp0=runif(nseason,0,1),

Rsigma.siteint=runif(1,1,2),

logN.cat=log(Cmax),w.cat=Cabund1,C.a=rnorm(2,0.5,0.1),C.beta=rnorm(2,0,0.1),

Csigma.lp=runif(1,1,2),C.b=runif(1,-1,1),C.c=rnorm(2,0,0.1),

Cp0=runif(nseason,0,1),Csigma.siteint=runif(1,1,2))

}

parameters <- c('R.a','R.b','R.beta','mean.Nrabbit','mean.Rp',

'Rsigma.lp','Rsigma.siteint','Rlogitp0',

'R.fit','fit.Rnew','N.rabbit','ik.Rp',

'C.a','C.b','C.c','C.beta','mean.Ncat','mean.Cp',

'Csigma.lp','Csigma.siteint','Clogitp0',

'C.fit','fit.Cnew','N.cat','ik.Cp')

library(R2WinBUGS)

M1 <- bugs (data, inits, parameters, "mod1.txt",

n.thin=10,n.chains=3, n.burnin=400000,n.iter=420000,debug=F)

##################### GRAPHICAL OUTPUT #######################################

#Bayesian p values:

mean(M1$sims.list$fit.Cnew>M1$sims.list$C.fit)

mean(M1$mean$C.fit)/mean(M1$mean$fit.Cnew)

mean(M1$sims.list$fit.Rnew>M1$sims.list$R.fit)

mean(M1$mean$R.fit)/mean(M1$mean$fit.Rnew)

##############################################################################

####plotting cat and rabbit abundances per kilometre

Rmax1<- matrix(1, nrow=nroute, ncol=nseason)

Cmax1<- matrix(1, nrow=nroute, ncol=nseason)

for (i in 1:nroute){

for (k in 1:nseason){

Rmax1[i,k] <- max(R[i,,k])

Cmax1[i,k] <- max(C[i,,k])

}}

seasons<-seq(1,11,1)

ssns<-c(rep(c('nb','b'),5),'nb')

dates<-seq(1990,1995,1)

uRoute<-unique(maind$Route)

t.length <- matrix(NA,nroute,nseason)

for (k in 1:nseason){

for (i in 1:nroute){

t.length[i,k]<-maind$Length[maind$Route==uRoute[i]& maind$Cperiod==k][1]

}}

for (i in 1:nroute){

t.length[i, is.na(t.length[i,])]<- max(t.length[i,],na.rm=T)

}

catxkm<-matrix(NA,nrow=nroute,ncol=nseason)

rabbitxkm<-matrix(NA,nrow=nroute,ncol=nseason)

for (i in 1:nroute){

for (k in 1:nseason){

catxkm[i,k]<-M1$mean$N.cat[i,k]/t.length[i,k]

rabbitxkm[i,k]<-M1$mean$N.rabbit[i,k]/t.length[i,k]

}}

mean.catxkm<-rep(NA,times=nseason)

mean.rabbitxkm<-rep(NA,times=nseason)

for (k in 1:nseason){

mean.catxkm[k]<-mean(catxkm[,k])

mean.rabbitxkm[k]<-mean(rabbitxkm[,k])

}

par(oma=c(1,3,1,1),mar=c(5,5,4,2))

matplot(t(catxkm),type='l',ylim=c(0,9), col='grey',xaxt='n',

tcl=.4, bty='l',ylab='Cats / km ',cex.axis=1.2,cex.lab=1.8)

lines(mean.catxkm, lwd=3, col='black')

axis(side=1,at=seasons,tcl=.4, lab=ssns, cex.axis=1.2)

axis(side=1,tcl=0,cex.axis=1.2,at=c(1.5,3,5,7,9,10.5),lab=dates,padj=2)

mtext("a)", 3, adj=-.2, cex= 2, padj=1.2)

par(oma=c(1,3,1,1),mar=c(5,5,4,2))

matplot(t(rabbitxkm),type='l',ylim=c(0,120),col='grey',xaxt='n',

tcl=.4, bty='l',ylab='Rabbits / km',cex.axis=1.2,cex.lab=1.8)

lines(mean.rabbitxkm, lwd=3, col='black')

axis(side=1,at=seasons,tcl=.4, lab=ssns, cex.axis=1.2)

axis(side=1,tcl=0,cex.axis=1.2,at=c(1.5,3,5,7,9,10.5),lab=dates,padj=2)

mtext("b)", 3, adj=-.2, cex= 2, padj=1.2)

###############################################################################

######### plotting relationship between cats & rabbits:

par(oma=c(1,3,1,1),mar=c(5,5,4,2))

plot(t(log(M1$mean$N.rabbit[,breed[]])),t(log(M1$mean$N.cat[,nonbreed[2:6]])),

type='p',lwd=2,, main='', tcl=.4, cex.axis=1.5,cex.lab=2,bty='l',

xlab='log(rabbit abundance (t))',ylab='log(cat abundance (t+1))')

mtext("a)", 3, adj=-.2, cex= 2, padj=1.2)

par(oma=c(1,3,1,1),mar=c(5,5,4,2))

plot(t(log(M1$mean$N.rabbit[,nonbreed[1:5]])),t(log(M1$mean$N.cat[,breed[]])),

type='p',lwd=2, main='', tcl=.4, cex.axis=1.5,cex.lab=2,bty='l',

xlab='log(rabbit abundance (t))',ylab='log(cat abundance (t+1))')

mtext("b)", 3, adj=-.2, cex= 2, padj=1.2)

###############################################################################

###plotting relationship between detection probability and transect length:

`expit` <-

function(x){

exp(x)/(1+exp(x))

}

scltransect<- seq(-1.8,2.8,by=.1)

meanLength<-mean(maind$Length)

sdLength<-sd(maind$Length)

sclLength<- seq(from=-2, to=3, by=1)

tl.pnts<-seq(15,40,by=5)

scl.pnts<-rep(NA,length(tl.pnts))

for (i in 1:length(scl.pnts)){

scl.pnts[i]<-(tl.pnts[i]-meanLength)/sdLength

}

#for cats

meanCestp<-rep(NA,nroute)

meanCestp <- expit(mean(M1$mean$Clogitp0) + (mean(M1$sims.matrix[,'C.beta[1]'])*scltransect) +

(mean(M1$sims.matrix[,'C.beta[2]'])*scltransect^2))

par(oma=c(1,3,1,1),mar=c(5,5,4,2))

plot(scltransect,meanCestp,ylim=c(0,0.6),xlim=c(-2,3),type='l',lwd=3, bty='l',xaxt='n',

cex.lab=2, cex.axis=1.5 , ylab='Detection probability', tcl=.4, xlab='Transect length (km)' )

points(transect.length,M1$mean$ik.Cp)

axis(side=1,at=scl.pnts,lab=signif(tl.pnts,2),tcl=.4,cex.axis=1.5)

mtext("a)", 3, adj=-.19, cex= 2, padj=1.2)

#for rabbits

meanRestp<-rep(NA,nroute)

meanRestp <- expit(mean(M1$mean$Rlogitp0) + (mean(M1$sims.matrix[,'R.beta[1]'])*scltransect) +

(mean(M1$sims.matrix[,'R.beta[2]'])*scltransect^2))

par(oma=c(1,3,1,1),mar=c(5,5,4,2))

plot(scltransect,meanRestp,ylim=c(0.5,0.8),xlim=c(-2,3),type='l',lwd=3, bty='l',xaxt='n',

cex.lab=2, cex.axis=1.5 , ylab='Detection probability', tcl=.4, xlab='Transect length (km)' )

points(transect.length,M1$mean$ik.Rp)

axis(side=1,at=scl.pnts,lab=signif(tl.pnts,2),tcl=.4,cex.axis=1.5)

mtext("b)", 3, adj=-.19, cex= 2, padj=1.2)

##################### END OF CODE #############################################
